# The ABA receptor PYL9 together with PYL8 plays an important role in regulating lateral root growth

**DOI:** 10.1038/srep27177

**Published:** 2016-06-03

**Authors:** Lu Xing, Yang Zhao, Jinghui Gao, Chengbin Xiang, Jian-Kang Zhu

**Affiliations:** 1 Hefei National Laboratory for Physical Sciences at Microscale and School of Life Sciences, University of Science and Technology of China, Hefei, Anhui 230026, China; 2 Shanghai Center for Plant Stress Biology, Shanghai Institutes for Biological Sciences, Chinese Academy of Sciences, Shanghai 200032, China; 3 Department of Horticulture and Landscape Architecture, Purdue University, West Lafayette, IN 47907, USA; 4 College of Animal Science and Technology, Northwest A&F University, Yangling, Shaan’xi 712100, China

## Abstract

Abscisic acid is a phytohormone regulating plant growth, development and stress responses. PYR1/PYL/RCAR proteins are ABA receptors that function by inhibiting PP2Cs to activate SnRK2s, resulting in phosphorylation of ABFs and other effectors of ABA response pathways. Exogenous ABA induces growth quiescence of lateral roots, which is prolonged by knockout of the ABA receptor PYL8. Among the 14 members of PYR1/PYL/RCAR protein family, PYL9 is a close relative of PYL8. Here we show that knockout of both *PYL9* and *PYL8* resulted in a longer ABA-induced quiescence on lateral root growth and a reduced sensitivity to ABA on primary root growth and lateral root formation compared to knockout of *PYL8* alone. Induced overexpression of *PYL9* promoted the lateral root elongation in the presence of ABA. The prolonged quiescent phase of the *pyl8-1pyl9* double mutant was reversed by exogenous IAA. PYL9 may regulate auxin-responsive genes *in vivo* through direct interaction with MYB77 and MYB44. Thus, PYL9 and PYL8 are both responsible for recovery of lateral root from ABA inhibition via MYB transcription factors.

As sessile organisms, plants need a sophisticated regulatory network to survive unfavorable and changing environments. When the soil environment becomes unfavorable, root system is often the first to sense it. This involves phytohormones that act quickly and accurately.

The phytohormone auxin is tightly correlated with both primary and lateral root growth and development. Auxin is perceived by a small family of F-box proteins including TRANSPORT INHIBITOR RESPONSE 1 (TIR1). Auxin gradient, which forms a sink at the root apex and just below quiescent center (QC), provides essential information for cell division, polarity and cell fate^1^. Lateral root initiation requires PIN-FORMED 1 (PIN1) -dependent auxin transport. During lateral root initiation, auxin determines both its position and frequency^2^. After initiation, auxin gradient is also required for the correct patterning of lateral root primordium^3^. To facilitate lateral root primordium emergence, auxin modulates cell turgor in the outer tissue layers and in the primordium^4^, and induces the expression of cell wall remodeling enzymes^5^,^6^,^7^. Besides auxin, other phytohormones, such as cytokinin, gibberellin, brassinosteroids, abscisic acid and strigolactones, also function during root growth. However, auxin acts as an integrator to them and these phytohormones either regulates polar auxin transport (PAT) or regulates auxin responsive genes.

Abscisic acid (ABA) is an isoprenoid plant hormone and a main regulator of responses to biotic and abiotic stress^8^. ABA biosynthesis is one of the quickest responses of plants facing stresses and ABA, in turn, will trigger downstream ABA responsive gene expression^9^. Besides its function on stress responses, ABA also has a role in plant development and physiological processes, including seed development and dormancy, embryo morphogenesis and stomatal movement^10^. The core signaling pathway of ABA includes receptors, phosphatases and kinases. The 14-member family of START domain proteins known as PYR1/PYLs/RCARs, has been identified as intracellular ABA receptors^11^,^12^. PYR1/PYLs/RCARs bind to and inhibit type 2C protein phosphatases (PP2Cs) in an ABA-dependent manner, which in turn release the inhibition of PP2Cs on SNF1-related kinase 2 (SnRK2 kinases)^11^,^13^. SnRK2 kinases are activated by activation loop autophosphorylation^14^ and are the key nodes in ABA signaling pathway.

High concentrations of ABA inhibit both primary and lateral root growth^15^,^16^, while low concentrations of ABA are known to promote root growth^17^. Studies with ABA-deficient mutants indicate that ABA is also crucial for maintenance of root growth under water-stressed and normal growth conditions^18^,^19^. This is partially due to the promotion of stem cell maintenance, which is vital for root growth, by nanomolar concentrations of ABA in the root meristem^20^.

Previous studies found that ABA and auxin are integrated into each other’s pathways in many developmental processes. ABA regulates auxin responses in many aspects. Loss of function of *ABA-insensitive 3* leads to a reduction of auxin-induced lateral root initiation^21^. Overexpression of *ABA-insensitive 4 (ABI4)* impairs lateral root development by reducing the expression of the auxin-efflux transporter PINFORMED 1 (PIN1)^22^. Furthermore, *Auxin Response Factor 2 (ARF2)* is a transcriptional repressor involved in plant growth and directly regulates the homeodomain gene *HB33.* Altered auxin distribution in *arf2-101* and two *HB33* overexpressing lines in response to ABA treatment indicates that ABA and auxin might act synergistically in inhibiting root growth^23^. Furthermore, the *pyl8* mutant shows a reduced sensitivity to ABA-mediated primary root growth inhibition^24^. Besides its role in primary root, PYL8 was found to interact with MYB DOMAIN PROTEIN 77 (MYB77) and functions in auxin-mediated lateral root growth^25^.

Auxin, in turn, also regulates ABA responses in many ways. During seed germination, the expression of *ABI3* is controlled by auxin through the auxin response factors AUXIN RESPONSE FACTOR 10 and AUXIN RESPONSE FACTOR 16^26^. Some auxin responsive genes are also regulators of stress responses. Among them is MYB77 that acts as a coactivator with ARFs^27^. Auxin-responsive gene expression was greatly attenuated in *myb77* knockout mutants and *MYB77* overexpression lines mimicked the wild type treated with exogenous IAA^27^. In the subgroup 22 where *MYB77* belongs to, there are three other members: *AtMYB44*/*AtMYBR1, AtMYB70* and *AtMYB73*^28^. Previous research has suggested that MYB44 represses ABA signaling during drought and senescence by interacting with PYL8 and PYL9^29^,^30^. Thus, these MYB proteins are associated with both auxin and stress responses^31^.

The members of PYR1/PYL/RCAR family have distinct properties. For example, two orthologs AtPYL13 and OsPYL12 inhibit several clade A PP2Cs in an ABA-independent manner, and most importantly, OsPYL12 is unable to bind to ABA^32^,^33^,^34^. PYR1 and PYL1-2 are dimers in solution, while PYL4-10 are monomers^35^. The diversity of PYLs may contribute to the versatility of ABA signaling.

Here, we report a function of PYL9 in regulating lateral root growth upon ABA treatment, in a manner similar to that of PYL8. Previous studies found that PYL9/RCAR1-mediated ABA signaling pathway could be modulated by ROP11 during *in vitro* reconstitution of ABA signaling pathway in *Arabidopsis* protoplasts^36^ and PYL9 could interact with an R2R3 MYB transcription factor, MYB44^29^. We found that PYL9 acts together with PYL8 in regulating the recovery of lateral root growth from inhibition after ABA treatment. Our results suggest that both PYL8 and PYL9 are nodes of crosstalk between ABA and auxin, and their signal is transduced by a group of MYB transcription factors to regulate the lateral roots.

## Results

### The *pyl8-1pyl9* double mutant shows a reduced inhibition of primary root growth and lateral root formation to exogenous ABA compared to *pyl8*

**Figure 1:**
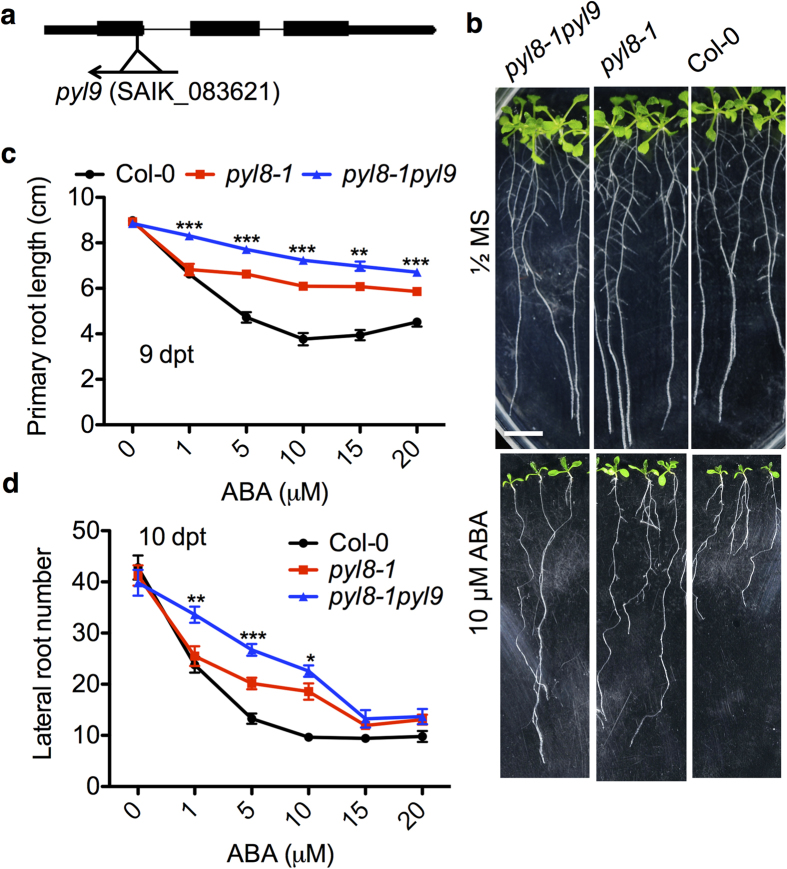
The primary root and lateral root formation of *pyl8-1pyl9* double mutant are more insensitive upon ABA treatment. (**a**) A schematic diagram of T-DNA insertions in the *PYL9* gene. The T-DNA insertion in the *pyl9* mutant is inserted in exon, which is presented as closed box. (**b**) Root architecture of Col-0, *pyl8-1* and *pyl8-1pyl9* mutants under ABA treatment. Root architecture of seedlings was documented at 9 dpt (days post transfer). Seedlings were transferred at 4 dpg (days post germination) to the control medium (1/2 MS, 1% sucrose) or medium with 10 μM ABA. Bar, 1 cm. (**c**,**d**) The primary root length and lateral root number of Col-0, *pyl8-1* and *pyl8-1pyl9* mutants were documented with different concentrations of ABA. The concentrations of ABA in the medium are as indicated. Error bars indicate s.e.m. (*n* = 25 seedlings, 5 independent experiments). Asterisks indicate comparison between *pyl8-1* and *pyl8-1pyl9.* *P < 0.05, **P < 0.01, ***P < 0.001, Student’s t test. P-values were adjusted for multiple comparisons by “Benjamini & Hochberg” method. Primary root length and lateral root number of *pyl8-1* (at 5 μM, 10 μM, 15 μM, 20 μM) and *pyl8-1pyl9* (at all ABA concentrations) were significantly larger than Col-0, P < 0.05.

ABA is a key factor in regulating root architecture under stress^16^,^20^. To dissect its role, we analyzed *Arabidopsis* mutants impaired in ABA signaling pathway, especially null mutants in ABA receptors. Previous studies demonstrated that PYL8 plays an important role in ABA signaling in roots and the work was focused on primary roots^24^. We found that in addition to regulating the growth of primary roots, PYL8 could also promote lateral root recovery from ABA inhibition^25^. Since PYL9 has a very high sequence identity with PYL8 and both of them have a relative high expression level in roots^37^, we obtained a T-DNA knock-out mutant of *pyl9* (SALK_083621) (Fig. 1a)^24^ and tested the root growth of *pyl9* under ABA treatment. *pyl9* mutant did not have an obvious lateral root growth phenotype on ABA medium (see Supplementary Fig. S1). We also quantified the primary root length and the lateral root number as well as average lateral root length both on the control medium and on ABA medium, still found no significant difference between *pyl9* and wild type (WT) (see Supplementary Fig. S1). PYL8 and PYL9 are in the same clade of the PYL phylogenetic tree^11^. We therefore analyzed the root growth of the *pyl8-1pyl9* double mutant^38^ (see Supplementary Fig. S2). Consistent with its visible phenotypes (Fig. 1b), the *pyl8-1pyl9* double mutant showed a faster primary root growth than the *py8-1* single mutant after treatment with ABA. On the 9^th^ day post transfer (dpt) to plates with ABA, the *pyl8-1pyl9* double mutant showed a longer primary root than *pyl8-1* single mutant at all concentrations of ABA tested (Fig. 1c). We also quantified the lateral root number of both mutants (Fig. 1d). On the 7 dpt to different concentrations of ABA, *pyl8-1pyl9* had more lateral roots than *pyl8-1* on 1, 5 and 10 μM ABA medium, and both of them differ significantly from WT at all concentrations except 1 μM ABA medium. Thus *pyl8-1pyl9* double mutant was less sensitive to ABA than *pyl8-1* single mutant in primary root growth and lateral root formation.

### Lateral root growth stays in a longer quiescence in *pyl8-1pyl9* double mutant

**Figure 2:**
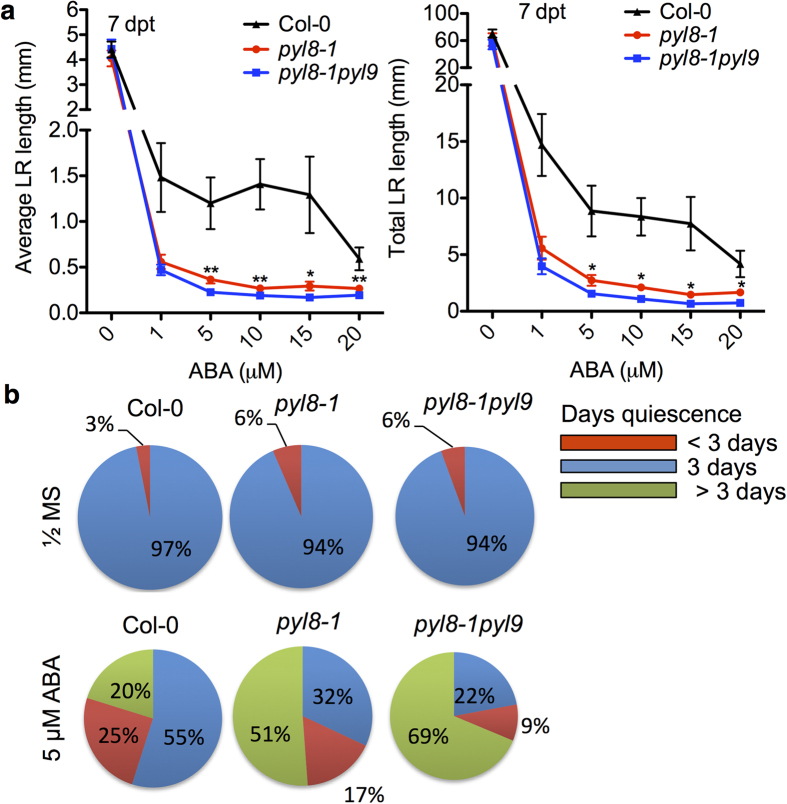
p*yl8-1pyl9* double mutant shows a prolonged quiescence phase compared to *pyl8-1* on ABA-containing medium. (**a**) Average and total lateral root lengths of Col-0, *pyl8-1* and *pyl8-1pyl9* mutants under different concentrations of ABA. Lateral root length of 7 dpt seedlings grown on ABA media were measured. The concentrations of ABA in the medium are as indicated. Error bars indicate s.e.m. (*n* = 25 seedlings, 5 independent experiments). *P < 0.05, **P < 0.01, Student’s t test. Asterisks indicate comparison between *pyl8-1* and *pyl8-1pyl9.* P-values were adjusted for multiple comparisons by “Benjamini & Hochberg” method. (**b**) Pie charts of the percentage of quiescent lateral roots for the indicated number of days on seedlings on control medium and medium with ABA (*n *= 25 seedlings per condition, 5 independent experiments). The proportion of quiescence greater than 3 days of *pyl8-1* is significantly less than that of *pyl8-1pyl9* (p-value < 0.05, one-tailed binomial test).

Besides primary root growth phenotypes, we have also observed that the *pyl8-1pyl9* double mutant had shorter lateral roots (Fig. 1b). Consistent with its visible phenotypes, the *pyl8-1pyl9* double mutant showed a slower lateral root growth than the *pyl8-1* single mutant after treatment with ABA. Lateral root growth was severely suppressed in both mutants exposed to ABA especially in the double mutant. Average lateral root length and total lateral root length were both decreased (Fig. 2a). The *pyl8* single mutant was reported to have a longer quiescent phase on ABA plates before recovery^25^. Our results here suggest that PYL9 may promote growth recovery of lateral root under ABA treatment together with PYL8. Lateral root with a length shorter than 0.5 mm is considered to be quiescent^39^. We found that the two mutants and the wild type had similar quiescent days on control medium. However both mutants have a prolonged quiescent phase than Col-0 under ABA treatment. Moreover, the *pyl8-1pyl9* double mutant had an even longer quiescent phase than the *py8-1* single mutant (Fig. 2b). This suggests that PYL9 functions together with PYL8 in promoting growth recovery of lateral roots under ABA treatment.

### IAA suppresses ABA-induced growth inhibition of lateral roots in *pyl8-1pyl9*

**Figure 3:**
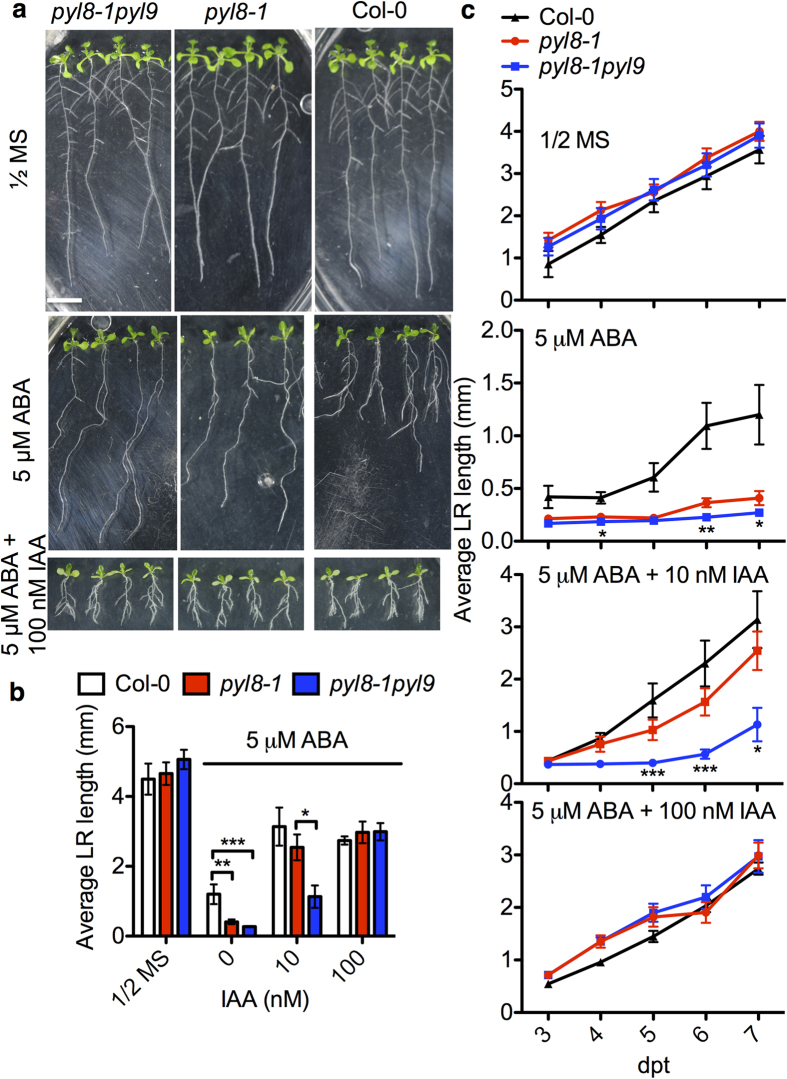
High exogenous IAA complements the lateral root growth defect of *pyl8-1pyl9* on ABA-containing medium. (**a**) Root architecture of Col-0, *pyl8-1* and *pyl8-1pyl9* mutants under ABA and ABA with IAA treatment. Root architecture of seedlings was documented at 7 dpt. Seedlings were transferred at 4 dpg to the control medium (1/2 MS, 1% sucrose) (upper panel) or medium supplemented with 5 μM ABA (middle panel) or 5 μM ABA and 100 nM IAA (bottom panel). Bar, 1 cm. (**b**) Average lateral root length of Col-0, *pyl8-1* and *pyl8-1pyl9* mutants grown with or without ABA treatment or ABA plus IAA treatment. Lateral root length of seedlings grown on ABA-containing medium was measured at 7 dpt. The concentrations of ABA and IAA in the medium are as indicated. Error bars indicate s.e.m. (*n* = 25 seedlings, 5 independent experiments). *P < 0.05, **P < 0.01, ***P < 0.001, Student’s t test. (**c**) The lateral root length of seedlings was measured at the indicated days after transfer to media with or without ABA or ABA plus IAA. Error bars indicate s.e.m. (*n* = 25 seedlings, 5 independent experiments). *P < 0.05, **P < 0.01, ***P < 0.001, Student’s t test. Asterisks indicate comparison between *pyl8-1* and *pyl8-1pyl9.*

Exogenous IAA can release growth inhibition of lateral roots under ABA treatment^25^. To determine whether this happens in the *pyl8-1pyl9* double mutant, we analyzed the lateral root growth of *pyl8-1pyl9* seedlings grown on ABA-containing medium supplemented with IAA (Fig. 3a). We found that the ABA-dependent lateral root growth suppression of *pyl8-1pyl9* was partially overcome by 10 nM IAA, and was fully rescued by 100 nM IAA (Fig. 3b). We also determined lateral root growth over the course of time. We found that both *pyl8-1* and *pyl8-1pyl9* mutants had a slower lateral root elongation than wild type under ABA treatment but not under control conditions. Exogenous application of 10 nM IAA rescued the ABA-dependent growth defects of lateral root in *pyl8-1* but not in *pyl8-1pyl9*. In contrast, in the presence of 100 nM IAA, the lateral root growth defects of both mutants were fully rescued (Fig. 3c). This means that IAA could also rescue the ABA-dependent inhibition of lateral root elongation caused by loss of *PYL8* and *PYL9*. However, the *pyl8-1pyl9* double mutant requires a higher concentration of IAA than *pyl8-1* single mutant. These results suggest that the lateral root growth defect of *pyl8-1pyl9* mutant may be caused by auxin deficiency.

### ABA-induced overexpression of *PYL9* alters both primary and lateral root growth

Since the function of PYL9 on root growth was relatively weaker than that of PYL8, we analyzed the root architecture of *PYL9* overexpression lines. The *PYL9* gene, under the control of *RD29A* promoter, was transformed into *Arabidopsis*. This *RD29A* gene was first discovered as a cold-induced gene. Later it was found to be responsive to not only cold but also dehydration and ABA^40^. Two homozygous *pRD29A:PYL9* transgenic lines with high *PYL9* expression were used for further analysis^38^. Both transgenic lines had a dramatic increase in *PYL9* expression compared to Col-0 wild type under ABA treatment (see Supplementary Fig. S3).

**Figure 4:**
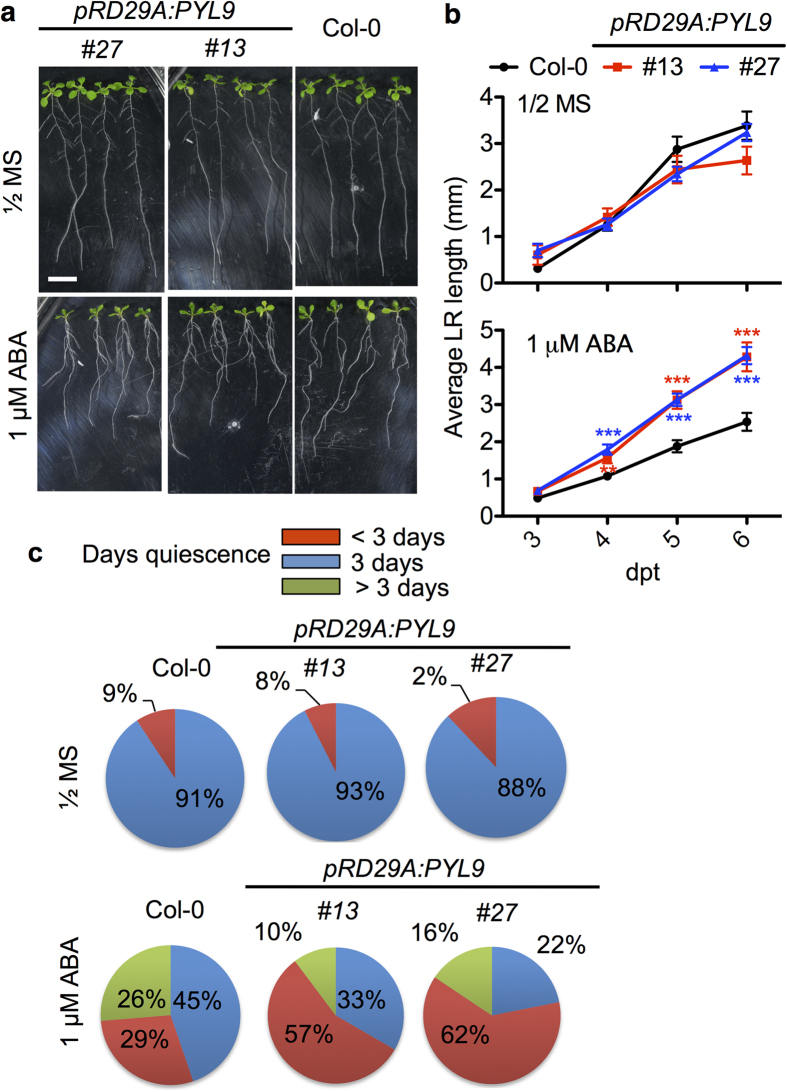
Induced expression of *PYL9* increases the average lateral root length and shortens the quiescent phase. (**a**) Root architecture of *pRD29A:PYL9#13, pRD29A:PYL9#27* and Col-0 under ABA treatment. Seedlings were transferred at 4 dpg to the control medium (1/2 MS, 1% sucrose) (upper panel) or medium supplemented with 1 μM ABA (bottom panel). Root architecture of seedlings was documented at 7 dpt. Bar, 1 cm. (**b**) The lateral root length of Col-0, *pRD29A:PYL9#13* and *pRD29A:PYL9#27* was measured at the indicated days after transfer to media supplemented with ABA. Seedlings were transferred at 4 dpg. Error bars indicate s.e.m. (*n* = 25 seedlings, 5 independent experiments). **P < 0.01, ***P < 0.001, Student’s t test. Asterisks indicate comparison between Col-0 and *pRD29A:PYL9#13,* Col-0 and *pRD29A:PYL9#27.* (**c**) Pie charts of the percentage of quiescent lateral roots for the indicated number of days on seedlings on control medium and medium with ABA (*n* = 25 seedlings, 5 independent experiments). The proportion of quiescence less than 3 days of Col-0 is significantly less than that of *pRD29A:PYL9#13* and *pRD29A:PYL9#27*, respectively (p-value < 0.001, one-tailed binomial test).

We transferred 4-day-old seedlings to both control medium and medium with 1 μM ABA (Fig. 4a). We monitored the lateral root length over time (Fig. 4b). The two transgenic lines did not have any obvious difference from wild type on control medium since the *PYL9* gene was not induced. When 1 μM ABA was applied, the transgenic lines produced longer lateral root than the wild type after 3 days and the difference was more significant over time (Fig. 4b). As loss of *PYL9* caused a prolonged quiescent phase, we wondered whether this increase in average lateral root length was due to a shortened quiescent phase. Under 1 μM ABA treatment, more than half of the lateral roots had a quiescent phase shorter than 3 days in both transgenic lines (Fig. 4c). Thus, overexpression of *PYL9* promotes lateral roots to escape from ABA-dependent inhibition.

**Figure 5:**
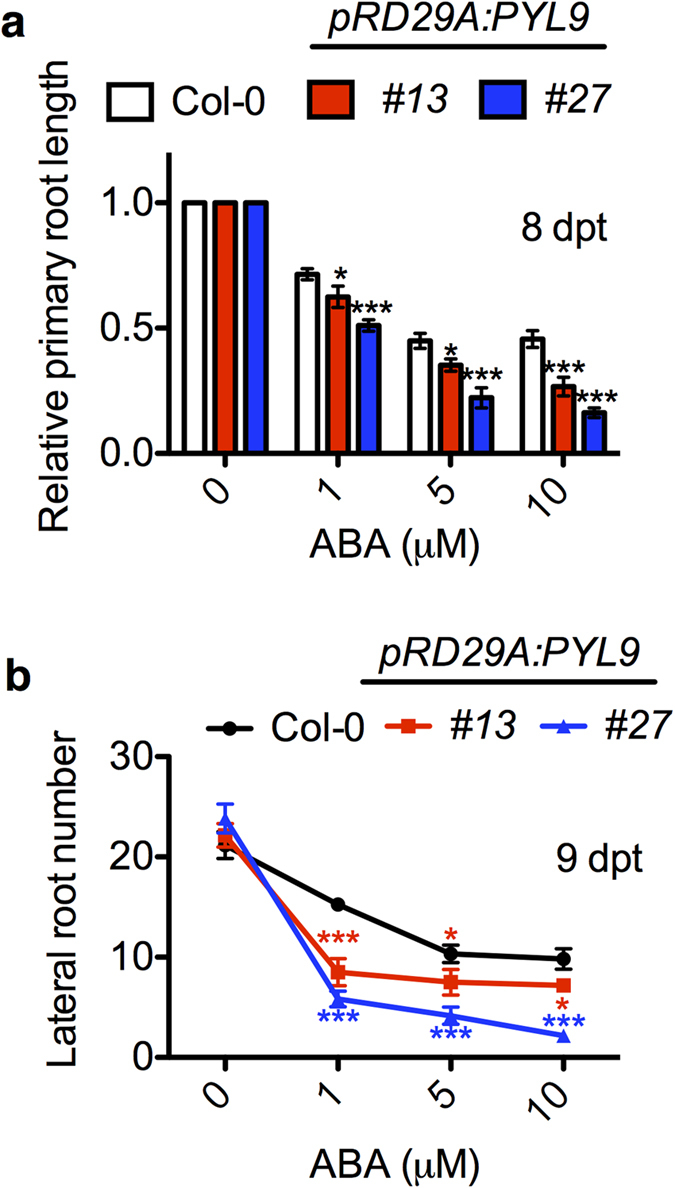
Induced expression of *PYL9* confers hypersensitivity of primary root and lateral root initiation. (**a**,**b**) Relative primary root length and lateral root number of Col-0, *pRD29A:PYL9#13* and *pRD29A:PYL9#27* under ABA treatment. The concentrations of ABA in the medium are as indicated. Measurements in **a** are expressed as a percentage of the length under 1/2 MS conditions. Error bars indicate s.e.m. (*n* = 25 seedlings, 5 independent experiments). *P < 0.05, ***P < 0.001, Student’s t test. Asterisks indicate comparison between Col-0 and *#13,* Col-0 and *#27.*

To test whether overexpression of ABA receptor *PYL9* conferred hypersensitivity to ABA in primary root growth, we analyzed root growth of *pRD29A:PYL9* transgenic lines with treatment of different concentrations of ABA. After transferring to medium supplemented with ABA and growing for a week, the primary root of transgenic lines showed an obvious reduction (Fig. 5a). After ABA treatment, both lines showed a significant reduction of lateral root number compared to the wild type (Fig. 5b). This suggests that PYL9 functions in the promotion of lateral root escaping from quiescence as well as the inhibition of primary root growth and lateral root formation under ABA treatment.

### PYL9 interacts with MYB transcription factors

**Figure 6:**
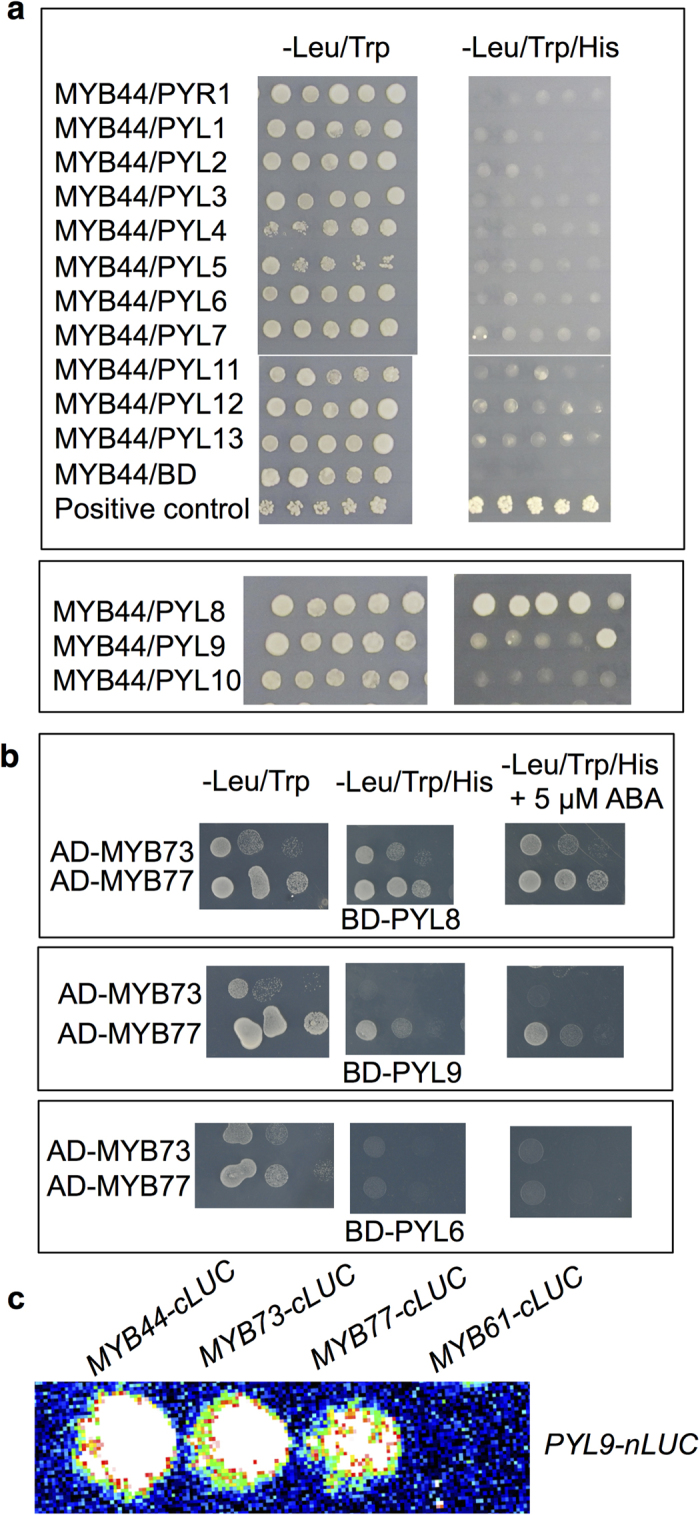
PYL9 interacts with MYB transcription factors. (**a**) PYLs interact with MYB44 in the yeast two-hybrid assay. PYLs fused to the GAL4-DNA-binding domain (BD) were used as bait. MYB44 fused to the GAL4-activating domain (AD) were used as preys. Interaction was determined by yeast growth on media lacking His, Leu and Trp. BD-PYL10 and AD-ABI1 was used as a positive control. (**b**) PYL9 interacts with MYBs in the yeast two-hybrid assay. PYL8 and MYB combinations were used as positive controls and PYL6 and MYBs were used as negative controls. Interaction was determined by growth on medium lacking His, Leu and Trp with or without 5 μM ABA. Dilutions (10^−1^, 10^−2^, and 10^−3^) of saturated cultures were spotted onto the plates, which were photographed after 5 days. (**c**) Co-transform of PYL9-nLUC with MYB44-cLUC, MYB73-cLUC, MYB77-cLUC and MYB61-cLUC in Col-0 wild-type protoplasts in LUC complementation assay.

Recently, interactions between PYLs and MYBs have been reported^25^,^29^,^30^. In our study, several different clones in each combination were tested on yeast growth medium deprived of Trp, His and Leu (Fig. 6a). All of these clones with MYB44/PYL8 had visible colonies, suggesting that PYL8 strongly interacts with MYB44. However, only one of these clones with MYB44/PYL9 had visible colonies (Fig. 6a), suggesting that PYL9 may weakly interact with MYB44. Other MYB proteins were also tested using multiple independent colonies. PYL6 was used as a negative control and had no interactions with any of the three tested MYBs (Fig. 6b, bottom panel). PYL8, as a positive control, had interactions with MYB77 and MYB73 (Fig. 6b, upper panel). Similar to clones with MYB44/PYL9, the minority of these independent clones with MYB77/PYL9 had visible colonies. However, we did not detect any interaction between MYB73 and PYL9 in the yeast two-hybrid assay (Fig. 6b, middle panel). These differences between our results and previous published results might be caused by the expression level of different plasmids. Li *et al.* used *pGADT7-MYB*s and the higher expressing *pGBKT7-PYL*s^29^, while Jaradat *et al.* used *pGADT7-PYL*s and the lower expressing *pGBT9-MYB*s^30^. We used *pGADT7-MYB*s and *pBD-GAL4-PYL*s. Our results and the previous findings suggest that PYL8 strongly interacts with MYBs, while PYL9 has a relatively weak interaction with MYBs.

To further analyze the interactions between PYL9 and MYBs in plant cells, we used the firefly luciferase (LUC) complementation assay in *Arabidopsis* protoplasts. PYL9 was fused to the N-terminal domain of firefly luciferase (LUC) and MYB proteins were fused to the C-terminal domain of LUC. We co-transformed PYL9-nLUC with the MYB-cLUC into *Arabidopsis* protoplasts (Fig. 6c). Coexpression of PYL9-nLUC with MYB44/73/77-cLUC, but not MYB61-cLUC produced measurable luciferase activity. These results suggest that PYL9 also interacts with some MYBs *in vivo*.

### PYL9 enhances the activity of MYBs in the presence of ABA and IAA

**Figure 7:**
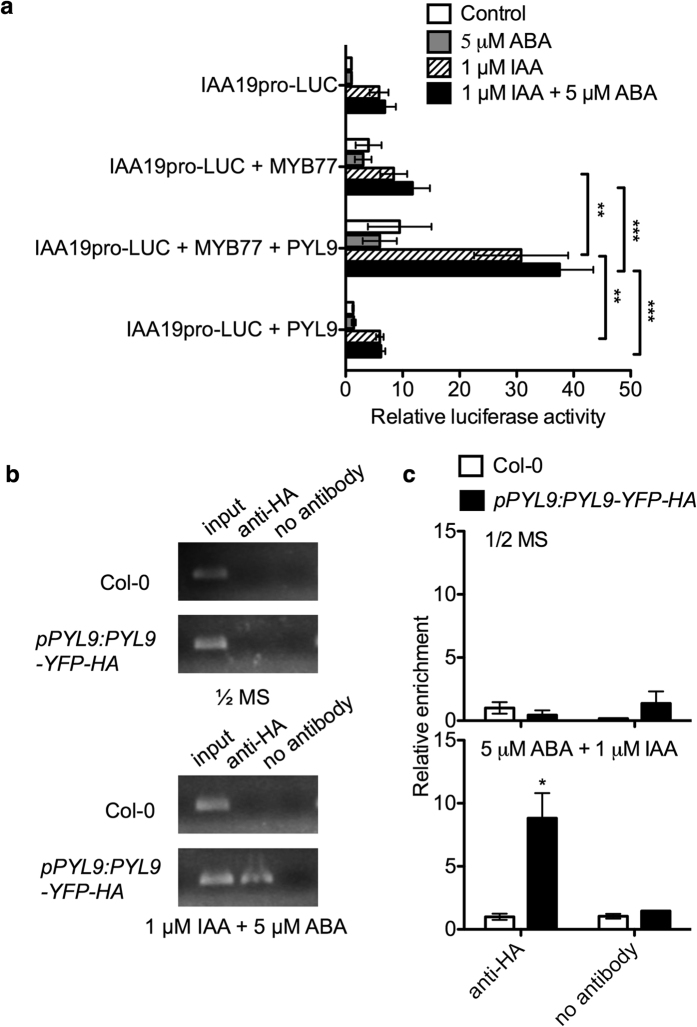
PYL9 directly regulates MYB77 transcriptional activity *in vivo.* (**a**) PYL9 enhances the ability of MYB77 to activate *IAA19* expression in Col-0 protoplasts. *PYL9*, *MYB77*, *IAA19-LUC* and *ZmUBQ-GUS* were co-expressed in protoplasts. *IAA19-LUC* was used as the auxin-responsive reporter. *ZmUBQ-GUS* was used as the internal control. After transfection, protoplasts were incubated for 12 h under light in the absence of hormone (open bars) or in the different combinations of 5 μM ABA and 1 μM IAA. Error bars indicate s.e.m. (*n* ≥ 3 experiments). **P < 0.01, ***P < 0.001, Student’s t test. (**b**,**c**) ChIP-PCR and ChIP-quantitative-PCR for *IAA7* promoter. The 500-bp region of the translational start of *IAA7* contains MBSI elements recognized by MYB77 and MYB44. *pPYL9:PYL9-YFP-HA* was treated with ABA and IAA before conducting ChIP assay. Col-0 was used as control. Numbers are folds compared to Col-0 adding anti-HA. Error bars indicate s.e.m. (*n* = 3 experiments). *P < 0.05, Student’s t test.

We asked whether the interaction with PYL9 might affect the regulation of downstream genes by MYBs. Previous studies showed that MYB77 could recognize *cis*-elements MBSI (CNGTTR) and MBSII (GTTAGTTA) and preferentially binds to the MBSI motif 25. *IAA19* is one of these downstream genes regulated by MYB77^27^. *pIAA19:LUC* was used as a reporter in the *Arabidopsis* protoplasts transient expression assay^25^. We co-expressed *PYL9*, *MYB77* with *pIAA19:LUC* in the protoplasts (Fig. 7a). As expected, the luciferase signal was induced by IAA and was enhanced by MYB77. Similar to PYL8, PYL9 enhanced the activity of MYB77 to increase the expression of *pIAA19:LUC* in the presence of IAA (Fig. 7a).

Previous studies demonstrated that PYL8 enhances the interaction between MYB77 and MBSI motifs *in vitro*^25^. To further understand whether PYL9 and MYB77 regulate their target genes as a protein complex, we used a transgenic line with tagged PYL9 driven by *PYL9* native promoter^38^. *pPYL9:PYL9-YFP-HA* was introduced into the quadruple mutant *pyr1pyl1pyl2pyl4* and this tagged line was used in chromatin immunoprecipitation assay (see Supplementary Fig. S4). We pre-incubated the seedlings with 5 μM ABA and 1 μM IAA for 5 hours, and the assay was performed as described^41^. We added extra EGS or ethylene glycol-bis (succinic acid N-hydroxysuccinimide ester) to strengthen the PYL9-MYBs interaction during cross-linking. Because MYB77 preferentially binds to MBSI, we filtered candidate genes using this criterion. We checked several DNA fragments that harbored this *cis*-element, including the promoter region of *IAA1, IAA7, IAA17, IAA19* and *HAT2.* Different primer pairs were designed flanking this motif. The ChIP-PCR result showed that the promoter region of *IAA7* was enriched with PYL9-YFP-HA after treatment of IAA and ABA (Fig. 7b). The ChIP-quantitative-PCR result showed nearly 10-fold enrichment in *pPYL9:PYL9-YFP-HA* transgenic lines than in the wild type negative control after the treatment of ABA and IAA. While in the control, there was no such enrichment (Fig. 7b,c). The motif in this promoter fragment was CTGTTG, belonging to MBSI. We also found another CTGTTG in the promoter of *HAT2* but this one did not show enrichment in ChIP-quantitative-PCR. This might be due to the position of the motif. The one in *IAA7* is located around 500 bp upstream from the start codon, however, the one in *HAT2* is located within 100 bp from its start codon. This suggests that the transcription complex of PYL9 and MYB proteins may bind to promoters around 500 bp from where transcription starts. This *in vivo* evidence strongly supports the hypothesis that PYL9 regulates the transcriptional activity of MYB77 or other MYB proteins directly *in vivo* and participates in the auxin signal pathway.

## Discussion

ABA and auxin are two important phytohormones; one is well known for its function under stress, and the other one is well known for its growth-inducing activity. Our study here reveals a crosstalk node between them in regulating root growth.

Roots provide a tight connection between plants and the soil environment, and are often the first to sense the soil environment. Root systems of ABA-deficient mutants like *aba2-1* and *aba3-1* are less affected than wild type seedlings upon osmotic stress^42^. ABA at low concentrations has been reported to promote root growth^17^. The endogenous ABA controls root architecture both in the presence and absence of osmotic stress through maintaining meristem in dormancy and inhibiting QC division and suppressing the differentiation of stem cells in the primary root^20^. High concentrations of ABA are well known to inhibit the growth of both primary and lateral root (Fig. 1c,d). ABA has a much stronger effect on lateral root than on the primary root, suggesting that different signaling mechanisms in the two types of roots^39^. In our study, the lateral root number in all the tested ABA concentrations showed a decrease compared to control medium (Fig. 1d,b). What’s more, the lateral root elongation is more sensitive to ABA than the inhibition of seed germination and this reversible growth arrest occurs at a specific developmental stage, that is right after the lateral root emergence with a length less than 0.5 mm^16^. As shown in Fig. 2b, longer quiescence was observed on ABA-containing medium and this might be due to a dormant state of lateral roots. Although ABA is well known for its function in seed dormancy and growth inhibition, different ABA sensitivities suggest different pathways in lateral roots compared with other tissues.

Salt stress induces a quiescence phase in post emergence lateral root growth and then recovery takes place several days later, which involves genes of ABA biosynthesis, signaling and transcription regulation^39^. PYL8 is responsible for this quiescence upon ABA treatment^25^. PYL9 has a 77% amino acid sequence identity with PYL8 and particularly high GUS activity can be detected in stele cells of *pPYL9:GUS* transgenic plants^24^. Although *pyl9* single mutant did not show obvious difference from wild type on ABA medium (see Supplementary Fig. S1), *pyl8-1pyl9* double mutant had an even longer primary root and more lateral roots than *pyl8-1* on ABA medium (Fig. 1c,d). The induced overexpression of *PYL9* upon ABA treatment led to a shorter primary root and fewer lateral roots (Fig. 5a,b). These results suggest that PYL9 functions in the repression of lateral root formation and primary root elongation by ABA. PYL9 is a functional ABA receptor^11^ and the ABA signal perceived by it and other PYLs is passed down eventually to *ABA-RESPONSIVE ELEMENT (ABRE)-BINDING FACTOR (ABF)* proteins. And overexpression of *ABF2* confers hypersensitivity to ABA in primary root^43^. Other mutants impaired in ABA signaling pathway have also been tested including *abi1-1, pyr1/pyl1/2/4* and *snrk2.2/3/6*^25^. All these ABA-resistant mutants showed a reduced repression of primary root growth and lateral root formation upon ABA treatment^25^ and this correlates with our results in *pyl8-1pyl9* double mutant. However, these ABA-resistant mutants have shorter quiescence compared to WT on ABA medium, which is opposite to our *pyl8-1pyl9* double mutant. This suggests a different pathway involving PYL9. Recently, interactions between PYLs and MYBs have been reported^25^,^29^,^30^. PYL8 strongly interacts with the MYBs^25^,^30^. Further studies found that PYL9 and probably PYL7 also interact with these MYBs^29^, which is different from the previous published result^30^. Our results also showed that PYL9 interacted with MYB77 and MYB44 (Fig. 6), however in our Y2H assay there was no obvious interaction between PYL9 and MYB73 (Fig. 6b), which is different from previous reports^29^. This is probably due to different yeast systems and plasmids. MYB77 is a positive regulator of lateral root growth and its interaction with ARF7 suggests that MYB77 may function through auxin signal transduction pathway^27^. The lateral root growth of *myb77* mutants is more sensitive upon ABA treatment and exogenous IAA could reverse this like it could reverse in *pyl8-1* and *pyl8-1pyl9*^25^. So the interaction between PYL9 and MYB77 suggests they may function together. MYB77 preferentially recognizes *cis*-elements MBSI (CNGTTR) and previous EMSA data showed that PYL8 protein enhances MYB77 binding to MBSI in an ABA-dependent manner^25^,^27^. MYB44 has a clear preference for MBSII (GTTAGTTA) type but still binds to MBSI^44^. Our chromatin immunoprecipitation assay indicated that anti-HA antibody pulls down the protein complex of PYL9-MYB proteins (Fig. 7b,c). The exogenous IAA and ABA enhanced their interaction and the effect on the downstream gene promoters^25^ (Fig. 7). Quantitative PCR showed that one fragment of *IAA7* promoter, containing MBSI motif, has a huge enrichment (Fig. 7b,c). IAA7 is a member of the Aux/IAA protein family, which is induced by auxin^45^. Besides PYL8 and PYL9, PYL7 is also an interacting protein of MYB44 in Y2H assays^29^. This implies that more PYLs might be involved in the PYL-MYB pathway.

Auxin and ABA are widely accepted as regulators of root growth. These two phytohormones have a lot of interactions and previous studies have revealed that auxin could potentiate ABA response in roots^46^. Unlike in *p35S:VP1* where ABA fully inhibits auxin induced lateral root initiation^47^, the prolonged quiescence phase of *pyl8-1* on ABA medium was shortened by application of IAA^25^. Osmotic stress represses lateral root development which could be overcome by auxin^42^, however exogenous ABA represses lateral root formation, that is the initiation of lateral root primordia, which could not be rescued by auxin^16^. In our study, the quiescence in *pyl8-1* and *pyl8-1pyl9* was overcome by application of IAA (Fig. 3). What’s more, the severe defects in the double mutant require more IAA to be rescued (Fig. 3b,c). Whether auxin rescues or not might be dependent on the state of lateral roots and these activities might be mediated by different mechanisms. Lateral root primordia initiation is highly dependent on auxin^48^, however in our study, we only considered the lateral roots that are already formed when we transferred seedlings to ABA medium.

The function of PYL9 and MYB77 complex is dependent on IAA treatment. In *Arabidopsis* protoplasts, the reporter (*pIAA19:LUC*) showed a strong signal when PYL9 was co-transformed with MYB77 in the presence of IAA (Fig. 7a). In transgenic seedlings, PYL9 can pull down auxin responsive promoter fragments in the presence of IAA and ABA (Fig. 7b,c). Besides, PYL9 itself is a functional ABA receptor. Taken together, this suggests that PYL9 connects ABA and auxin signaling pathway. MYB77 modulates auxin response by forming a heterodimer with ARF7 and their double mutant *myb77-2arf7 (myb77-2nph4-1)* has even smaller lateral root density than *nph4-1,* which is already smaller than wild type and *myb77-2*^27^. In *myb77* mutants, auxin-responsive genes are attenuated while they are increased in over-expression lines^27^. Similar results were obtained in *p35S:MYB44* transgenic lines where auxin-responsive genes are increased, suggesting that salt response and auxin signaling cross-talk at the transcriptional level^31^. This suggests that MYB77 and MYB44 might have some overlapping functions in regulating auxin-responsive genes. *IAA7,* which might be the target of PYL9-MYB77 complex, is also a key component regulating ABA and auxin-dependent post-embryonic growth^49^. It is suppressed when ABA represses embryonic axis elongation by potentiating auxin signaling in the elongation zone^49^.

PYL9 binds type 2C protein phosphatases and was found to interact with R2R3 MYB proteins recently. Our results show that PYL9 not just binds to MYB transcription factors, but also regulates their transcriptional activity, and that this is a point of convergence between ABA and auxin pathways.

## Methods

### Plant materials and growth condition

The *pyl8-1* (SAIL_1269_A02)^24^, *pyl9* (SALK_083621)^24^, *pyr1pyl1/2/4*^11^ quadruple mutant were in Col-0 background. The *pyl8-1pyl9* double mutant was obtained by crossing *pyl8-1* with *pyl9.* The *pRD29A::PYL9* construct was transformed into Col-0, and *pPYL9::PYL9-YFP-HA* construct was transformed into *pyr1pyl1/2/4* by *Agrobacterium tumefaciens-*mediated floral-dip transformation.

Seeds were surface-sterilized for 8 min in 10% bleach and then rinsed in sterile deionized water for four times. Sterilized seeds were grown on 0.6% Phytagel (Sigma) or 0.8% agar media containing 1/2 MS nutrients (catalog no. M524, PhytoTechnology Laboratories), 1% sucrose adjusted to pH 5.7 (control media), and kept at 4 °C for 2 days. Seedlings were grown vertically before transfer to control media or media supplemented with the indicated concentrations of ABA (Sigma, A1049) or IAA (Sigma, I2886). Seedlings were grown at 22 °C under 16-h light/8-h dark cycles.

For protoplast analysis, seedlings were grown on Jiffy 7 peat soil (42 mm Pellets) in a Percival chamber with a relatively short photoperiod (12 hours of light at 23 °C, 12 hours of dark at 20 °C) under low light (about 100 μE m^−2^ s^−1^) and 50 to 70% relative humidity under well-watered conditions.

### Phenotype analysis

The primary root length was measured from the first day after transfer. The results of lateral root were observed using HIROX KH-7700 digital microscope with MX-5040RZ lens. The total and average lateral root lengths of individual plants were quantified by summing or averaging the lengths of all the lateral roots on each plant. Lateral roots shorter than 0.5 mm were categorized as quiescent^39^.

### Plasmid construction

The *pIAA19-LUC* and *pGADT7-MYBs* constructs were generated as described^25^. The *pBD-GAL4-PYLs* constructs were generated as described^11^.

### Yeast two-hybrid assay

Yeast two-hybrid assays were performed as described^25^. PYLs fused to the GAL4 DNA binding domain were used as baits. MYB44, MYB73 and MYB77 fused to the GAL4-activating domain were used as preys. Interaction was determined by growth assay on media lacking His or His and Ade with or without 5 μM ABA. Dilutions (10^−1^, 10^−2^ and 10^−3^) of saturated cultures were spotted onto the plates and photographed after 5 days.

### Transient expression assay in *Arabidopsis*

Assays for transient expression in protoplasts were performed as described^25^. All steps were at room temperature. *pIAA19::LUC* was used as the auxin-responsive reporter. *ZmUBQ-GUS* was used as the internal control. After transforming, protoplasts were incubated in washing and incubation solution (0.5 M mannitol, 20 mM KCl, 4 mM MES, pH 5.7) with or without ABA and IAA at the indicated concentrations for 12 hours of light and LUC luminescence was measured with a plate reader (Wallac VICTOR2 plate reader). LUC complementation assays were performed as described^25^.

### Chromatin immuoprecipitation assay

Ten-day-old transgenic seedlings and anti-HA antibodies (1:100 for ChIP assay, HA-Tag, 26D11, Mouse mAb, M20003, Abmart, Shanghai, China) were used for ChIP experiments^50^. Briefly, the transgenic seedlings were ground into a fine powder with liquid nitrogen and resuspended in nuclei isolation buffer. 1.5 mM EGS was added into the nuclei isolation buffer for protein-protein cross-linking. The nucleus were then collected by centrifugation and resuspended with nuclei lysis buffer. The resuspended chromatin was sonicated to fragments with various sizes (250 bp–1 kb) subsequently. PYL9-HA-YFP-MYBs protein complex was precipitated from input DNA with or without anti-HA antibodies. Protein A agarose beads (Millipore, USA) were added into the incubation mixture for additional 2 h at 4 °C. The immune complexes were eluted from the washed protein A beads. The DNA was purified with phenol/chloroform (1:1, v/v) and precipitated. The purified DNA and input DNA were used as templates. The enrichment of DNA fragments was determined by quantitative PCR with specific primers.

## Additional Information

**How to cite this article**: Xing, L. *et al.* The ABA receptor PYL9 together with PYL8 plays an important role in regulating lateral root growth. *Sci. Rep.* **6**, 27177; doi: 10.1038/srep27177 (2016).

## Supplementary Material

Supplementary Information
