# Supplementary material for: The ABA receptor PYL9 together with PYL8 plays an important role in regulating lateral root growth
Source: Sci Rep. 2016 Jun 3;6:27177. doi: 10.1038/srep27177 (PMC4891660; doi:10.1038/srep27177)
Supplement: Supplementary Information [file srep27177-s1.doc]

# The ABA receptor PYL9 together with PYL8 plays an important role in regulating lateral root growth

Lu Xing^1,3,+^, Yang Zhao^2,3,+^, Jinghui Gao^3,4,+^, Chengbin Xiang^1,*^, Jian-Kang Zhu^2,3,*^

^1^Hefei National Laboratory for Physical Sciences at Microscale and School of Life Sciences, University of Science and Technology of China, Hefei, Anhui 230026, China.

^2^Shanghai Center for Plant Stress Biology, Shanghai Institutes for Biological Sciences, Chinese Academy of Sciences, Shanghai 200032, China.

^3^Department of Horticulture and Landscape Architecture, Purdue University, West Lafayette, IN 47907, USA.

^4^College of Animal Science and Technology, Northwest A&F University, Yangling, Shaan'xi 712100, China.

^+^These authors contributed equally to this work.

Correspondence and requests for materials should be addressed to C.X. (email: xiangcb@ustc.edu.cn) or J.K.Z. (email: jkzhu@sibs.ac.cn)


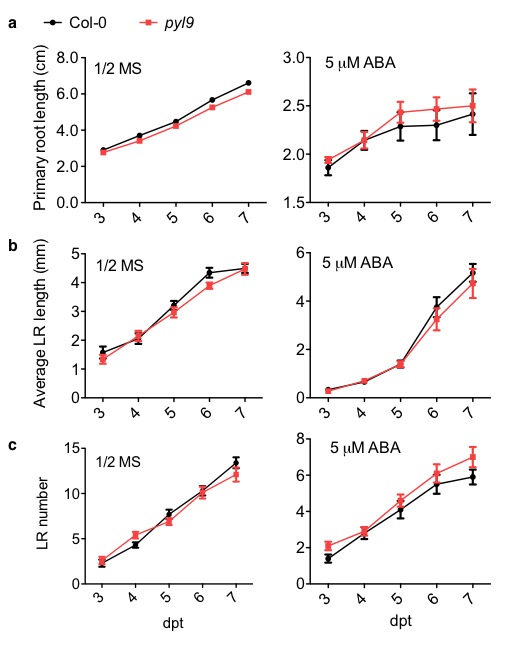


**Supplementary Figure S1. *pyl9* single mutant has no obvious phenotype on ABA-containing medium.** (**a, b** and **c**) Primary root length, average lateral root length and lateral root number of Col-0 and *pyl9* were measured at the indicated days after transfer to media supplemented with ABA. Seedlings were transferred at 4 dpg (days post germination). Error bars indicate s.e.m. (*n* = 10 seedlings).


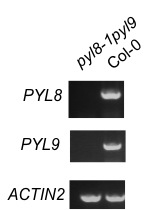


**Supplementary Figure S2. RT-PCR of *pyl8-1pyl9* and Col-0.** Plants were grown on 1/2 MS medium vertically for 10 days and 0.1g (fresh weight) whole seedlings were used to extract RNA and transcribed into cDNA for further analysis.


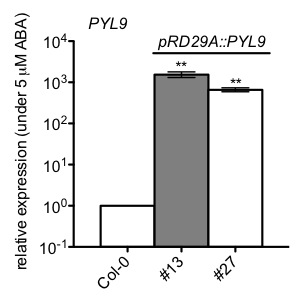


**Supplementary Figure S3. *PYL9* expression level in *pRD29A::PYL9* under 5 µM ABA treatment.** 10-day-old seedlings were subjected to 1/2 MS plus 5 µM ABA liquid medium and 4 hours incubation. Error bars indicate s.e.m. (*n* = 3). **P <0.01, Student’s t test. The expression of *PYL9* was set at zero.


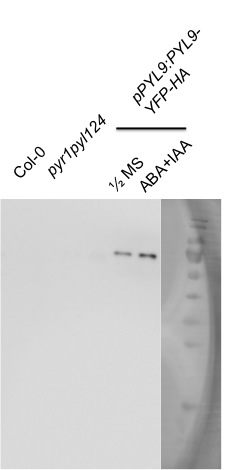


**Supplementary Figure S4. Detection of PYL9-YFP-HA protein in a sample purified from 10-day-old seedlings of *pPYL9::PYL9-YFP-HA* expressing lines.** Total protein was extracted from 10-day-old seedlings grown on 1/2 MS medium. Anti-HA antibody was used to pull down the PYL9-YFP-HA protein and the tagged protein was detected with anti-GFP antibody in Western Blot.
